# Editorial: Progresses in the Drug Treatment of Chronic Cardiopulmonary Diseases

**DOI:** 10.3389/fphar.2022.910212

**Published:** 2022-05-19

**Authors:** Xiaohui Li, Djuro Kosanovic, Xiao-Jian Wang, Yunshan Cao

**Affiliations:** ^1^ Department of Pharmacology, Xiangya School of Pharmaceutical Science, Central South University, Changsha, China; ^2^ Department of Pulmonology, I.M. Sechenov First Moscow State Medical University (Sechenov University), Moscow, Russia; ^3^ Key Laboratory of Pulmonary Vascular Medicine, State Key Laboratory of Cardiovascular Disease, Fuwai Hospital, National Center for Cardiovascular Diseases, Chinese Academy Medical Sciences and Peking Union Medical College, Beijing, China; ^4^ Department of Cardiology, Gansu Provincial Hospital, Lanzhou, China

**Keywords:** chronic cardiopulmonary diseases, pulmonary embolism, pulmonary fibrosis, pulmonary hypertension, drug development

We are delighted to announce the publication of the special issue with the topic “Progresses in the Drug Treatment of Chronic Cardiopulmonary Diseases”. One hundred and thirty two authors made their contributions to this issue and the editors finally assembled 18 full-length articles. The scale of accepted papers is wide covering from basic research to translational medicine and clinical studies. The content includes molecular mechanisms, meta-analysis, candidate compounds, metabolomics and bioinformatics. In our opinion these studies show advanced and useful information in this field, provide new insights into understanding of pathological mechanisms or identification of new therapeutical targets in Chronic Cardiopulmonary Diseases (CCD).

The authors paid attention to the pathophysiological mechanisms and biomarkers in challenging diseases including idiopathic pulmonary fibrosis (IPF), pulmonary hypertension (PH) and pulmonary embolism (PE). For example, it is reported that Sirtuin (Sirt2) may participate in the development of IPF *via* regulating the drosophila mothers against decapentaplegic2/3 (Smad2/3) pathway (Gong et al.). Interferon regulatory factor 9 (IRF9) facilitates pulmonary artery smooth muscle cells proliferation by regulating prohibitin1 (PHB1) expression and protein kinase B (AKT) signaling pathway to affect mitochondrial function during the development of PH (Chen et al.). MicroRNAs with pleiotropic effects have great potential as biomarkers and therapeutic targets for CCD (Condorelli et al., 2014; Saha et al., 2019; Agbu and Carthew, 2021)**.** Luo et al. summarized the literature on miRNA functions in PE, suggesting that miRNA combined with traditional biomarkers or miRNA signatures generated from microchips may be a good predictive tool for PE occurrence and prognosis (Luo et al.).

There are three articles summarizing the advances in PH, pulmonary thromboembolism (PTE) and chronic obstructive pulmonary disease (COPD) and obstructive sleep apnea (OSAS) overlap syndrome (OS). The nitric oxide (NO) pathway is one of the key pathways underlying the pathophysiology of PH (Lau et al., 2017; Galiè et al., 2019; Gorenflo and Ziesenitz, 2021), Tettey et al. suggested that several new substances targeting on NO pathway show a promising potential to represent future alternatives. The current knowledge indicates that COPD and OSAS patients may be at higher risk to develop the cardiovascular diseases. Collection and analysis of clinical data demonstrated that patients with OS possessed deteriorating baseline characteristics and an increased prevalence of cardiovascular diseases, among them the heart failure and PH, compared to the patients with COPD or OSAS alone (Tang et al.). This is a clear reminder for clinicians to be alerted that OS patients have elevated risk of cardiovascular diseases and that early detection and adequate treatment are crucial for these individuals. Furthermore, it is known that PTE is an important cause of death in the context of cardiovascular diseases (Duffett et al., 2020; Papamatheakis et al., 2020; Piazza, 2020). Following this line, Cao et al. emphasized an overlooked condition, *in situ* pulmonary artery thrombosis and compared the risk factors, the common and specific pathogenic mechanisms underlying PTE, *in situ* pulmonary artery thrombosis, and chronic thromboembolic pulmonary hypertension (CTEPH), coining a new concept of pulmonary artery thrombotic disease and facilitating our understanding of pathogenesis, differential diagnosis, and personalised therapeutics of the three pulmonary artery thrombotic diseases (Cao et al.).

Metabolomics and bioinformatics are increasingly used approaches to identify the differential features between the states of health and disease (Meikle et al., 2014; Ussher et al., 2016; Meyer and Calfee, 2017; Wishart, 2019; Schiano et al., 2020). Scientists from China, Netherlands, Switzerland and Germany together identified a metabolic profile that distinguished pulmonary artery smooth muscle cells from hypoxia-treated and control groups. This project discovered six hypoxia-induced metabolism associated hub genes in response to hypoxia (He et al.), which would lead to the better understanding of the profound molecular mechanisms in hypoxic PH and provide valuable clues to researchers who would follow these emerging genes in the future. Connective tissue disease-associated pulmonary arterial hypertension (CTD-PAH) related gene sets were obtained through text mining by Tan et al. Furthermore, the intersection of gene sets was analyzed for functional enrichment through the DAVID and the STRING was used for the determination of the protein-protein interaction network of the overlapping genes and the significant gene modules. As the outcome, the enriched candidate genes were finally analyzed by Drug Gene Interaction database to find 13 drugs targeting six genes which may potentially result in beneficial therapeutic effects against the severe disease, such as CTD-PAH (Tan et al.).

Meta-analysis is still popular approach with regard to the clinical research of CCD, especially for assessment of the therapeutical effects or adverse events (Schiattarella et al., 2017). Articles discuss the role of inhaled corticosteroids (ICSs) and anti-interleukin-5 (IL-5) therapies in COPD (Chen et al.; Zhang et al.). In addition, clinical practice of sodium-glucose cotransporter 2 inhibitors (SGLT2is) is summarized with regard to the prevention of cardiovascular and respiratory dysfunctions (Yin et al.; Bhatia et al., 2021). Some articles discuss compounds derived from plant extracts or medicinal chemistry research as novel potential treatments for CCD. For example, magnolol isolated from Magnolia Officinalis, Luteolin, Naringin usually extracted from tomatoes and citrus fruits and Sanguinarine, a benzophenanthridine alkaloid obtained primarily from the bloodroot plant were investigated in several pre-clinical experimental models of PH and right ventricle hypertrophy (Fu et al.; Zuo et al.; Wu et al.; Fan et al.). Results of these studies strongly suggested the promising pharmacological effects and revealed the underlying mechanisms of above mentioned natural compounds. One study also indicated the new clinical application of Xuezhikang, an extract of cholestin, which consists of lovastatin, phytosterols and isoflavones and it was previously used to reduce the serum lipid concentrations. Results showed the potential therapeutic effect of Xuezhikang on PH patients with low serum high-density lipoprotein cholesterol (HDL-C) levels (Cao et al.). In the past, we became aware of the existence of significant limitations with regard to the monotherapy, and subsequently the clinical drug combinations were continuously investigated (Lajoie et al., 2016; Savale et al., 2020; Hoeper et al., 2004). Following this paradigm, Shi et al. found that therapy with rapamycin and low dose imatinib together achieved more beneficial effects as compared to the respective monotherapies in order to prevent the PH development. Importantly, new compound and novel therapeutical strategy were developed by Li et al., this compound is HLQ2g which was designed to share the structure of Riociguat and also harbor the anti-fibrosis unit. Basically, it consists of pyrazolpyridine ring (anti-fibrosis functional group) and pyrimidine ring (stimulating soluble guanylate cyclase (sGC) functional group) (Hu et al., 2022). Bifunctional HLQ2g might represent a new pharmacological strategy for PH.

In conclusion, it is clear that despite intensive research efforts to identify and develop new therapies over the past two decades, CCD remain a group of diseases characterized with high morbidity and mortality. The urgent clinical needs are still not properly addressed. Research in this field has become a hot and key venue, waiting for exciting new findings emerging from basic and clinical investigations into the pathophysiology and precise molecular pathobiology of CCD. We strongly call for more attention to CCD and the use of more cutting-edge technology to develop better treatment options.
